# Selection of single blastocysts for fresh transfer via standard morphology assessment alone and with array CGH for good prognosis IVF patients: results from a randomized pilot study

**DOI:** 10.1186/1755-8166-5-24

**Published:** 2012-05-02

**Authors:** Zhihong Yang, Jiaen Liu, Gary S Collins, Shala A Salem, Xiaohong Liu, Sarah S Lyle, Alison C Peck, E Scott Sills, Rifaat D Salem

**Affiliations:** 1 Division of Reproductive Endocrinology Research, Pacific Reproductive Center, Torrance, CA, 90505, USA; 2 IVF Division, Beijing Jia En De Yun Hospital, Beijing, 100083, People's Republic of China; 3 Centre for Statistics in Medicine, Wolfson College Annexe, University of Oxford, Oxford, UK

## Abstract

**Background:**

Single embryo transfer (SET) remains underutilized as a strategy to reduce multiple gestation risk in IVF, and its overall lower pregnancy rate underscores the need for improved techniques to select one embryo for fresh transfer. This study explored use of comprehensive chromosomal screening by array CGH (aCGH) to provide this advantage and improve pregnancy rate from SET.

**Methods:**

First-time IVF patients with a good prognosis (age <35, no prior miscarriage) and normal karyotype seeking elective SET were prospectively randomized into two groups: In Group A, embryos were selected on the basis of morphology and comprehensive chromosomal screening via aCGH (from d5 trophectoderm biopsy) while Group B embryos were assessed by morphology only. All patients had a single fresh blastocyst transferred on d6. Laboratory parameters and clinical pregnancy rates were compared between the two groups.

**Results:**

For patients in Group A (*n* = 55), 425 blastocysts were biopsied and analyzed via aCGH (7.7 blastocysts/patient). Aneuploidy was detected in 191/425 (44.9%) of blastocysts in this group. For patients in Group B (*n* = 48), 389 blastocysts were microscopically examined (8.1 blastocysts/patient). Clinical pregnancy rate was significantly higher in the morphology + aCGH group compared to the morphology-only group (70.9 and 45.8%, respectively; *p* = 0.017); ongoing pregnancy rate for Groups A and B were 69.1 vs. 41.7%, respectively (*p* = 0.009). There were no twin pregnancies.

**Conclusion:**

Although aCGH followed by frozen embryo transfer has been used to screen at risk embryos (e.g., known parental chromosomal translocation or history of recurrent pregnancy loss), this is the first description of aCGH fully integrated with a clinical IVF program to select single blastocysts for fresh SET in good prognosis patients. The observed aneuploidy rate (44.9%) among biopsied blastocysts highlights the inherent imprecision of SET when conventional morphology is used alone. Embryos randomized to the aCGH group implanted with greater efficiency, resulted in clinical pregnancy more often, and yielded a lower miscarriage rate than those selected without aCGH. Additional studies are needed to verify our pilot data and confirm a role for on-site, rapid aCGH for IVF patients contemplating fresh SET.

## Background

Multiple gestation represents the most significant complication of assisted reproductive treatment (ART). Single embryo transfer (SET), either elective or mandatory, has been advocated as an effective means to avoid multiple gestation following IVF [1-3]. Despite a welcome trend in increased acceptance and utilization of elective SET treatment in some groups [4], most IVF cycles continue to involve two or more embryos for transfer. When SET is done, selection of the single embryo or blastocyst for transfer is typically done on the basis of morphology [5,6]. However, since acceptable morphology alone cannot negate the potential for chromosomal error in the selected embryo, the transfer of one apparently “normal looking” embryo carries considerable risk [7]. Aneuploidy is the most common abnormality in human embryos derived from IVF [8-15], a problem that contributes substantially to poor IVF outcomes [16]. As other investigators have noted, screening embryos by fluorescence in situ hybridization (FISH) was a reasonable response to this challenge, but the approach was limited because it failed to screen all chromosomes at the same time [17-21]. Conventional comparative genomic hybridization (CGH) has been used for comprehensive screening of aneuploidy for oocytes and embryos [19,22-25] with cryopreservation of embryo(s) from which the biopsy was derived. When results became available, frozen embryo transfer (FET) was subsequently arranged so that only euploid embryo(s) were transferred.

At present, there is no consensus on the best way to determine the competency of the embryonic genome during IVF. Both single nucleotide polymorphism (SNP) array and array CGH (aCGH) have been validated as accurate methods to achieve comprehensive chromosome screening when biopsy is performed on d3 for fresh transfer on d5 [26-30]. The difference in mosaicism between embryos at d3 and d5 has led to a preference for biopsy at the blastocyst stage when mosaicism is reduced [31-33]. When combined with trophectoderm biopsy and blastocyst vitrification, SNP microarray has resulted in high implantation rate and low miscarriage rates for some IVF patients [31]. However, experience is limited with aCGH to select a single euploid blastocyst for fresh transfer in the absence of known chromosomal diagnosis. In this pilot study, we evaluated a rapid, on-site aCGH application to select a single euploid blastocyst for fresh transfer in good prognosis patients <35 yrs of age, who were undergoing a first IVF attempt.

## Methods

### Patient sample

Following IRB approval, patients undergoing IVF at our programs in Beijing and Los Angeles were offered enrollment in this prospective, single-blind, pilot interventional study to compare embryo assessment by conventional microscopy alone or with array comparative genomic hybridization (aCGH) performed on trophectoderm. Written informed consent was obtained from all study participants and all received pre-treatment counseling in anticipation of possible incorporation of aCGH in their IVF treatment. Patients were eligible for this study if (female) age was <35 yrs, if there was a history of regular ovulation, if etiology of infertility was tubal factor or male factor (or both), and if no prior IVF treatment had been initiated. Additionally, all study subjects were required to have a normal intrauterine contour (confirmed by hysteroscopy), both ovaries intact, basal serum FSH and estradiol on d2-3 at <10 IU/l and <60 pg/ml, respectively. IVF patients whose treatment incorporated donor gametes or frozen/thawed embryos were excluded. A random number table was used to determine patients in vitro laboratory management strategy as either (1) traditional morphology assessment plus aCGH (Group A, *n* = 55), or (2) conventional morphology assessment only (Group B, *n* = 48). Patients (but not laboratory or clinical staff) were blinded with regard to their randomization group. The two cohorts were mutually exclusive, and no study patient had embryos assigned to both laboratory groups.

### Ovarian stimulation and fertilization

Before commencing gonadotropin therapy patients underwent transvaginal ultrasound evaluation with re-measurement of serum FSH, LH and estradiol on d3 of the index cycle. Pituitary downregulation was achieved with GnRH-agonist administered on d21 of the cycle immediately preceding treatment, as previously described [33]. Periodic transvaginal ultrasound and serum estradiol measurements were used to track follicular growth and thickness of endometrial lining. When ≥3 follicles reached 19 mm mean diameter, periovulatory hCG was administered by subcutaneous injection of recombinant hCG (250 μg Ovidrel®, Merck Serono; Geneva, Switzerland) with oocyte retrieval performed under transvaginal ultrasound guidance 35-36 h later. Following removal of all cumulus cells, ICSI was performed and normal fertilization was verified 16-18 h after injection by presence of two pronuclei and two polar bodies.

### Embryo culture and trophectoderm biopsy

All embryos were cultured in sequential media (Vitrolife; Göteborg, Sweden) to blastocyst stage. On d3 when embryos were at the 6–8 cell stage, a noncontact 1.48 μ diode laser (OCTAX Microscience GmbH; Bruckberg, Germany) was used to create a circular 6-9 μ diameter opening in the zona pellucida. For embryos randomized to the aCGH group, this breach enabled biopsy of trophectoderm (TE) on d5 rapidly. Between 3–5 herniated TE cells were gently aspirated by pipette and, when necessary, freed from the blastocyst by application of several laser pulses. Harvested TE cells were washed in PBS and placed within a PCR tube with 2.5 μl 1x PBS as previously described [34]. A uniform assisted hatching methodology was used for all embryos irrespective of subsequent TE biopsy or conventional microscopic assessment alone.

### aCGH protocol

Whole genome amplification was performed on-site using the SurePlex DNA amplification system (BlueGnome Ltd; Cambridge, UK) in accordance with manufacturer’s guidelines, as described elsewhere [34,35]. Briefly, samples and control DNA (8 μl for each) were labeled with Cy3 and Cy5 fluorophores (BlueGnome Ltd; Cambridge, UK). Labeling time was approximately 3 h with DNA resuspended in dexsulphate hybridization buffer and hybridized overnight under cover slides. After washing 1x 10 min in saline sodium citrate (SSC)/0.05% Tween-20 at room temperature, an additional irrigation in SSC 1x 10 min was completed at room temperature. Slides were washed in SSC 1x 5 min at 60°C and again for 1 min at room temperature (in SSC). Vacuum centrifuge was used to dry microarray slides over 3 min, followed by laser scanning at 10 μm (Agilent Technologies; Santa Clara, USA). Microarray data were analyzed with BlueFuse software (BlueGnome, Cambridge, UK) for chromatin loss or gain across all 24 chromosomes. Aberrations were considered non-artifact if ≥15 probes deviated from normal limits as defined by the 24Sure platform. The published accuracy rate for this aCGH technique when applied to TE cells is 95% [35].

### Blastocyst grading and selection for transfer

In both aCGH and control groups, blastocysts were graded [36] on a 1 to 6 scale determined by degree of expansion and hatching status, as follows: Grade 1 (early blastocyst): blastocoele <1/2 of total embryo volume; Grade 2 (intermediate blastocyst): blastocoele ≥1/2 of total embryo volume; Grade 3 (full blastocyst): blastocoele fully occupies the embryo; Grade 4 (expanded blastocyst): blastocoele is larger than early blastocyst and zona pellucida (ZP) demonstrates thinning; Grade 5 (hatching blastocyst): herniation of trophectoderm cells from the ZP; and Grade 6 (hatched blastocyst): blastocyst has escaped the ZP. For blastocysts at Grades 3 to 6, the inner cell mass (ICM) and trophectoderm (TE) were also graded. The ICM was graded as follows: A (many ICM cells packed together tightly); B (several ICM cells grouped loosely) and C (very few ICM cells). TE was graded as follows: A (many TE cells forming multiple epithelial layers); B (few TE cells consisting of a loose epithelium) and C (very few large TE cells).

Fresh SET was performed on the morning of d6 under direct ultrasound guidance for all patients. For embryos in the aCGH group only one euploid blastocyst was selected for transfer, based on data from the aCGH analysis. When multiple euploid blastocysts were available (as determined by aCGH), the best grade euploid blastocyst was selected for transfer. Any surplus euploid blastocysts were vitrified for later use [34]. In the non-aCGH (control) group, a single blastocyst was selected for fresh transfer based on morphological criteria only (e.g., no aCGH evaluation). The surplus blastocysts with good morphology (grade 3BB or above) were vitrified for future FET cycles.

### Outcome measures and statistical analysis

Clinical pregnancy rates were tabulated and compared for IVF patients in both groups. Clinical pregnancy was defined as an intrauterine gestational sac containing one embryo which demonstrated cardiac action with rate ≥110/min [37], and pregnancies at ≥20 weeks of gestation were classified at on-going. Differences between groups were assessed by Chi-squared and Fisher’s exact tests. A difference of *p* < 0.05 was considered statistically significant.

## Results

**Figure 1:**
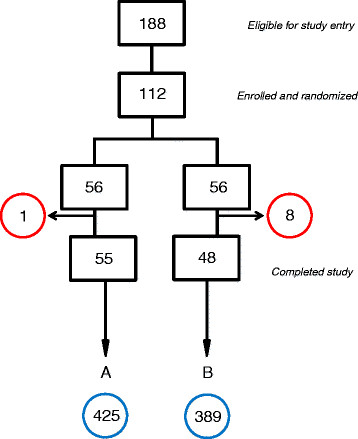
**Schematic for patients randomized either to embryo assessment by standard morphology plus aCGH (A) or morphology alone (B).** Withdrawals, deferrals and drop-outs for each group are circled in red. The total number of blastocysts associated with each group is circled in blue.

During the four-month study interval, a total of 188 IVF patients met inclusion criteria and 112 volunteered for enrollment (59.6%). Fifty six patients were randomized to each group. Of these, some patients did not initiate IVF due to failure to complete mandatory pre-IVF testing, they rescheduled their IVF, or they withdrew from treatment for personal reasons (see Figure 1). For Group A (morphology + aCGH) and Group B (morphology only) 55 and 48 IVF patients completed the study, respectively. The clinical and demographic features of the two groups were similar, as summarized in Table 1. There were no cancellations or complications for any patient in either study group.

**Table 1:** Characteristics of patients whose embryos were randomized to assessment by morphology with aCGH (Group A) and blastocyst morphology only (Group B)

	Group A (*n* = 55)	Group B (*n* = 48)
Age (yrs)	31.2 ± 2.5	31.5 ± 2.7
Total oocytes retrieved	19.5 ± 8.2	19.3 ± 8.1
MII (mature) oocytes	16.6 ± 7.8	16.3 ± 7.6
Oocytes fertilized (2*pn*)	13.1 ± 6.7	12.8 ± 6.4
Day 3 embryos	12.9 ± 1.8	12.6 ± 1.9
Day 5 blastocysts	8.3 ± 2.1	8.1 ± 2.4

*Notes*: Total number of blastocysts in Group [A] and [B] were 457 and 389, respectively. aCGH = array comparative genomic hybridization, MII = metaphase II, 2*pn* = two pronuclei. All data reported as mean ± SD. There was no significant difference between groups (*p* > 0.05) in any category.

For patients in Group A, 425 of 457 blastocysts were biopsied and analyzed via array CGH (7.7 blastocysts/patient). Biopsy could not be completed for 32 blastocysts due to poor morphology or because they degenerated after biopsy. This evaluation revealed aneuploidy in 191/425 (44.9%) of blastocysts. ‘No signal’ due to amplification failure occurred in 8 blastocysts. Among aneuploid blastocysts, 68/191 (35.6%) had single chromosome loss (monosomy) and 20.9% displayed single chromosome gain (trisomy). Approximately 43% of aneuploid blastocysts were chromosomally abnormal due to a severe, compound genetic defect where two or more chromosomes were affected (see Table 2). While chromosomal abnormalities were detected in all chromosomes, disruptions involving chromosomes 15, 16, 21, 22 and X were most frequently observed. Errors of chromosomes 4 and 6 were relatively uncommon. All patients in Group A had at least one euploid blastocyst available for transfer on d6. For patients in Group B, 389 blastocysts were microscopically examined (8.1 blastocysts/patient).

**Table 2:** **Detail of aCGH results derived from aneuploid blastocysts (*n*** **= 191) in Group A**

	*n* (%)
Single chromosome loss (monosomy)	68 (35.6)
Single chromosome gain (trisomy)	40 (20.9)
Dual chromosomal abnormality	55 (28.8)
Complex chromosomal abnormality	28 (14.7)

A single embryo was selected for transfer to all patients on d6. As shown in Table 3, the observed ongoing pregnancy rate was significantly higher in the morphology + aCGH group compared to the morphology-only group (69.1 vs. 41.7%, respectively; *p* = 0.009). A significant difference in clinical pregnancy rate was also noted between the two study groups (70.9 vs. 45.8%, respectively; *p* = 0.017). There were no twin pregnancies identified in either group. A low miscarriage rate was noted for all study patients, although this was somewhat lower in the morphology + aCCH group than for the morphology-only group (2.6 vs. 9.1%, respectively; *p* = 0.597, by Fisher’s exact test).

**Table 3:** Comparison of laboratory findings and clinical outcome among IVF patients undergoing SET with embryo assessment by aCGH + morphology (Group A) and blastocyst morphology alone (Group B)

	A	B	*p*
Fresh blastocyst transfer *according to morphology assessment:*	55 (100)	48 (100)	
*Grade 5/6*	31 (56.4)	28 (58.3)	
*Grade 4*	21 (38.2)	19 (39.6)	0.677^a^
*Grade 3*	3 (5.4)	1 (2.1)	
Clinical pregnancy	39 (70.9)	22 (45.8)	0.017^a^
Ongoing pregnancy (≥20wks GA)	38 (69.1)	20 (41.7)	0.009^a^
Missed abortion	1 (2.6)	2 (9.1)	0.597^b^

*Notes*: All data reported as *n* (%). SET = single embryo transfer; aCGH = array comparative genomic hybridization; GA = gestational age ^a^ by Chi-squared test ^b^ by Fisher’s exact test.

## Discussion

Delivery of a healthy singleton live birth is the target outcome for all infertility treatment. Although elective SET has emerged as the best answer to reduce the multiple gestation rate in IVF, uncertainty about the technique itself, low patient awareness of the process, lack of a favorable reimbursement system, and inferior cryopreservation success rates have hindered the uptake of this approach [38]. The value of promoting SET was recently underscored by a population-based cohort study of IVF outcomes where cerebral palsy (CP) incidence was noted among 1042 IVF singletons born after SET in Denmark [39]. Only one of those children received a CP diagnosis, compared with 21 CP diagnoses among IVF singletons born after two or more embryo transfers [39]. In Canada, efforts to mandate SET gained support from a multi-year review showing how this change in IVF practice would prevent infant deaths and reduce serious complications associated with multiple gestations [40]. Researchers found 17% of all NICU admissions—82 infants from 44 multiple gestations—resulted from assisted fertility treatments, and most NICU admissions (75 of 82 infants) were twins or triplets whose mothers used IVF to become pregnant. Among those 75 babies there were 6 deaths, and 5 more developed severe intraventricular hemorrhage [40].

Given this background, IVF patients should be encouraged to consider elective SET during pre-treatment counseling. Except for Sweden and Belgium [41,42], all other jurisdictions allow the decision for number of embryos for transfer to be made by doctor and patient, so the role of the reproductive endocrinologist in this process is vital [38]. How the choice to have elective SET is communicated has been shown to be an important influencing factor as this choice is made [43]. Yet in many clinics, if SET is offered at all, it is the patient herself who requests this option. Confidence in chance of success after SET, younger patient age, and first IVF treatment appear to favor a patient asking for SET [44]. We support the basic criteria for elective SET as proposed by others [45], including age <37 yrs, at least two good quality embryos available (3–5 cells on d2 or 6–9 cells on d3; <20% fragmentation and no multinucleate blastomeres), and no more than one previous failed treatment cycle. Among Australian IVF patients, preference for a healthy singleton pregnancy was predictive for elective SET, but perception of risk of multiple gestation was not [44]. Reporting on IVF patients in Ireland, Walsh *et al.*[46] investigated pre-treatment anxiety about twins and no association with patient age was observed. When presented with the option of SET, good prognosis IVF patients in Ireland agreed with this approach [47].

So why hasn’t elective SET found wider application in clinical IVF practice? Low pregnancy rates after fresh SET [48-51] have limited its acceptance, but this criticism of elective SET may be offset when cumulative outcome with subsequent frozen embryo transfer (FET) cycles is considered [52-55]. To be sure, more IVF patients would request elective SET if the success rate approached that following a two embryo transfer [56]. It is therefore understandable for both patients and clinicians to view elective SET with skepticism unless significant refinements in fresh embryo assessment come forward to facilitate the selection of competent embryos.

The current study extends prior research where aCGH was used for IVF patients with a known chromosomal rearrangement [29,35], and is the first to apply this technology to embryos from young, good prognosis patients undertaking IVF for the first time. Because SET is more frequently requested by IVF patients with a favorable prognosis [47], and since in this setting the clinical urgency to identify the best single embryo for transfer is maximal, our hypothesis developed this clinical problem into a therapeutic solution where aCGH figured prominently. Incorporating aCGH within an IVF clinic not only promises improved reproductive competency of each embryo at fresh transfer, it also offers important ploidy information regarding any supernumary (non-transferred) embryos which may be cryopreserved for later use. At our center, integrating aCGH with the clinical IVF program was associated with the same extra cost typically charged for the more limited genetic assessment gained from 5-probe FISH—less than $3000. These considerations should be particularly welcome among patients and clinicians contemplating elective SET, but who hesitate to make decisions without the advantage of comprehensive chromosomal screening. Moreover, an integrated testing approach also removed the a priori requirement for material to be frozen and shipped off-site for testing, followed by arranging subsequent FET based on findings from aCGH performed remotely. We believe that patient stress was reduced by eliminating FET medications entirely, while also reducing overall IVF treatment time. How patients quantify the distinctions between fresh transfer and FET treatment regimes is the target of ongoing study.

**Figure 2:**
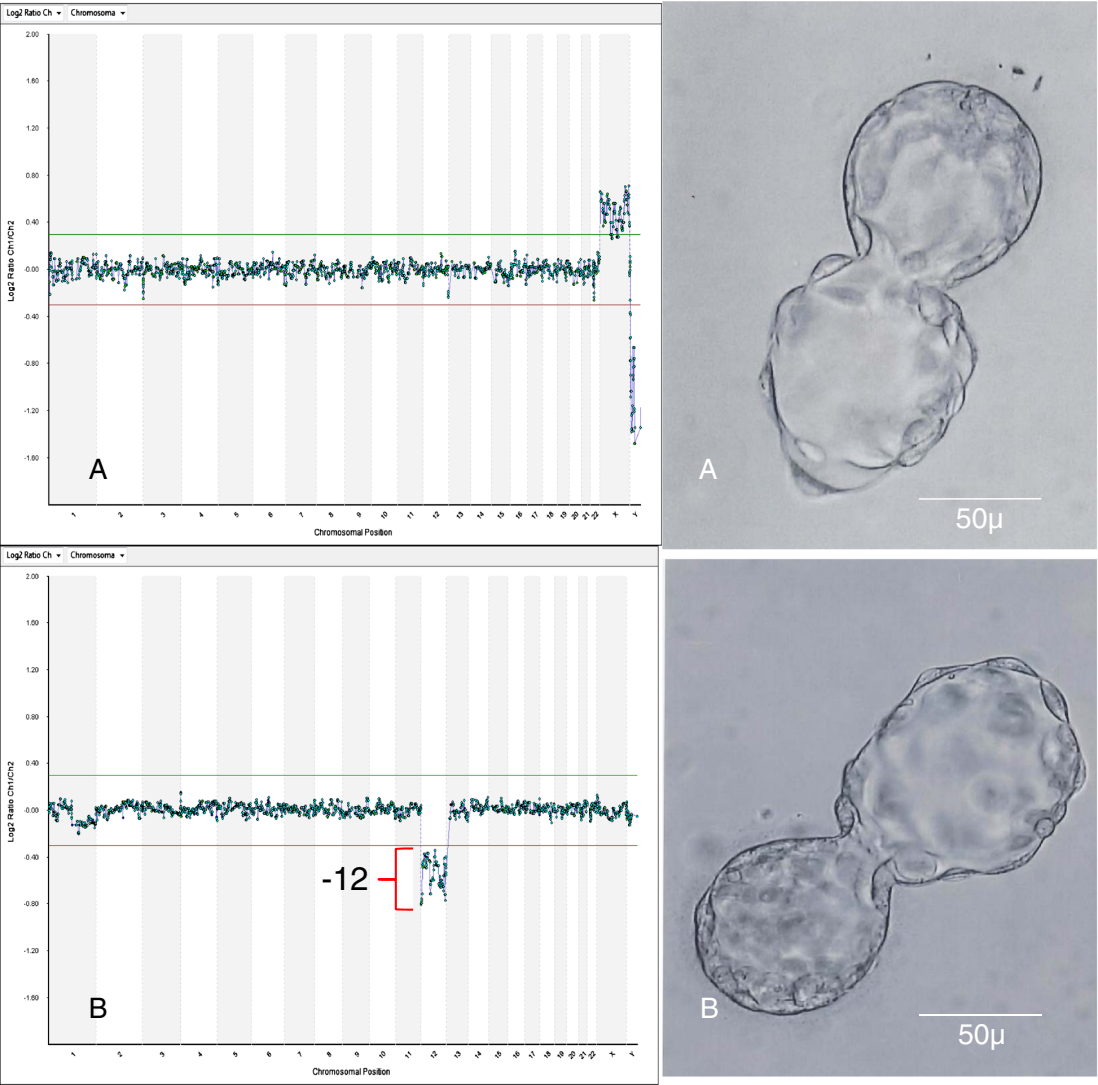
**Representative aCGH data obtained from human blastocysts via trophectoderm biopsy performed on post-fertilization day 5.** While standard microscopy confirmed good morphology (Grade 5AA) for both blastocysts, ploidy status was not uniform. Using aCGH to screen embryos before fresh transfer, normal chromosomal status (46,XX) was verified in A, but not in B (45,XY,-12).

Our research contributes new aCGH data on embryos from good-prognosis IVF patients, placing the limitations of standard embryo morphology in sharp relief. The extent of aneuploidy in early human embryos can be extensive [11,57,58] although this rate is typically lower in blastocysts [25]. Yet, the current study provides further evidence of substantial genetic abnormality in apparently normal blastocysts, including monosomy and complex aneuploidy [7,25,59]. Our data show conventional morphological criteria alone to be insufficiently accurate even for young, low-risk IVF patients (see Figure 2). Recent research on thawed blastocysts after SNP-based comprehensive chromosomal screening and vitrification has yielded similar results [60].

Several limitations of our investigation should be acknowledged. First, although elective SET brings distinct advantages for many IVF patients, the approach is not for everyone. Indiscriminate use of elective SET for patients with multiple failed cycles has been criticized as inferior to a two-embryo strategy [61], and the improved pregnancy rate noted here may not fully generalize to all IVF patients. Additionally, this pilot study was designed to use aCGH for selection of a single blastocyst for fresh transfer. It is possible that embryo assessment by conventional morphology inappropriately excludes euploid embryos from transfer although this question was outside the scope of our study. Hence, the relation between chromosomal integrity and morphological grades based on developmental stage, ICM and TE appearance, requires further investigation with a larger sample.

## Conclusion

In this pilot study, we have shown that the prospect of a successful IVF outcome with elective SET may be substantially lifted if aCGH testing is integrated with the clinical IVF program. The observed discordance between ploidy status and morphology means embryo selection without the benefit of information gained from aCGH would allow the transfer of a reproductively incompetent—albeit morphologically normal—embryo. Although these initial SET data are encouraging, a multi-center randomized clinical trial with a larger sample is planned to validate these preliminary findings.

## Competing interest

The authors declare that they have no competing interests.

## Authors’ contributions

ZY and JL conceived the research, designed the study, and directed the aCGH analysis. GSC was the chief statistician in charge of data analysis. SAS, XL, ACP, ESS, and RDS were the reproductive endocrinologists with oversight of the clinical program. SSL was the embryologist. ESS edited the manuscript and organized the revisions. All authors read and approved the final manuscript.
